# Lung Organoids in Smoking Research: Current Advances and Future Promises

**DOI:** 10.3390/biom12101463

**Published:** 2022-10-12

**Authors:** Hina Agraval, Hong Wei Chu

**Affiliations:** Department of Medicine, National Jewish Health, Denver, CO 80206, USA

**Keywords:** lung organoids, lung progenitors, stem cells, tobacco smoking, COPD

## Abstract

Tobacco smoking has been established to contribute to the pathogenesis of various respiratory diseases including chronic obstructive pulmonary disease (COPD), lung cancer, and asthma. However, major hurdles in mechanistic studies on the role of smoking in human lungs remain in part due to the lack of ex vivo experimental models and ambiguous data from animal models that can best recapitulate the architecture and pathophysiology of the human lung. Recent development of the lung organoid culture system has opened new avenues for respiratory disease research as organoids are proving to be a sophisticated ex vivo model that functionally and structurally mimics the human lungs better than other traditionally used models. This review will discuss how recent advances in lung organoid systems may help us better determine the injurious and immunological effect of smoking on human lungs and will provide some suggestions for future research directions.

## 1. Introduction

The human lung is one of the most critical and complex organs with more than 40 different types of cells that comprise epithelial cells, immune cells, endothelial cells, and stromal cells [1]. The airway epithelium is lined with different types of cells such as multi-ciliated cells and secretory cells (club and goblet cells) which produce a layer of liquid (mucus) that functions to moisten the air and offer an antimicrobial defense [2]. Lungs are constantly exposed to numerous airborne hazards (pollutants, cigarette smoke, bacteria, viruses, and other toxic agents) that can cause extensive damage to the lungs, resulting in progressive diseases, including asthma, chronic obstructive pulmonary disease (COPD), lung cancer, and respiratory infections. The remarkable regenerative capacity of the lung in response to the damage caused by airborne hazards is due to an impressive arsenal of defenses (e.g., defensins, lactoferrin, etc.) that lungs possess. How the lungs regenerate following an injury or disease has been a major focus of lung research [3]. Notably, recent studies have discovered several types of stem/progenitor cells that can proliferate and undergo phenotypic reprogramming during development or in response to injury [4].

Smoking is an established risk factor for several lung pathologies. Cigarette smoking is responsible for approximately one out of five deaths in the United States. It is also directly responsible for almost 90% of lung cancer and COPD-associated deaths [5]. For the past few decades, research groups working in inhalation toxicology have used numerous models to understand the disease pathophysiology and underlying mechanisms of smoking-induced lung damage. Cell-culture-based models in the form of immortalized cell lines and primary cells isolated from human lungs have been used to study lung development and disease pathogenesis, as they are easy to handle and cost-efficient when compared to other models such as in vivo models. However, cultures from primary cells are not suitable for long-term studies as they cannot be sub-cultured after a few passages, and the cultures from immortalized cells are genetically and phenotypically different from their in vivo counterparts. Air–liquid interface culture cannot completely mimic the naïve airway epithelium and the microenvironment control is also limited. On the other hand, species-specific variabilities are one of the major limiting factors for animal models used in lung research. Additionally, these models cannot mimic the cellular complexity of the human lungs [6,7,8]. Thus, there is a dire need for using recently developed models that can better recapitulate the in vivo lung disease pathophysiology associated with smoking. In this regard, lung organoids, due to their self-renewal and differentiation potential, have caught the attention of the research community to explore mechanisms and therapeutic approaches for cigarette-smoke-induced lung diseases.

In this review, we will focus on the organoids derived from mouse and human lung stem or organ-specific progenitor cells. Lung organoids developed in the past few years have been shown to imitate the lung development process and recapitulate the 3D structure and function of the lung [9]. Over the past few years, researchers have developed lung organoids from basal cells, adult stem cells (ASCs), induced pluripotent stem cells (iPSCs), and human pluripotent stem cells (hPSCs) [2]. This review will highlight the advances made in the development of lung organoids and how lung organoids can provide us insights into lung development, injury, repair, and disease pathophysiology. We will further discuss how lung organoids can be exploited as a novel and useful tool to study the effect of cigarette smoking on lung pathologies.

## 2. Cigarette Smoking and Lung Health

**Figure 1:**
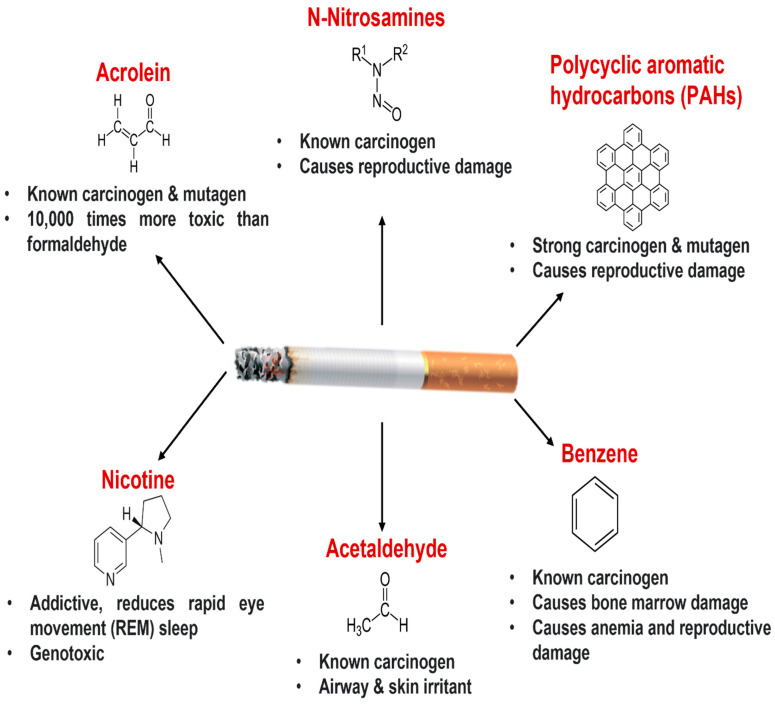
Cigarette smoke components and their pathological effects: Cigarette smoke contains more than 4000 chemicals that exert cytotoxic, mutagenic, and carcinogenic effects. Nicotine is the prime addictive ingredient of cigarette smoke which is genotoxic and has immunomodulatory effects [11]. Acrolein has carcinogenic and mutagenic potential in animal models (rats and mice) and humans [34,35]. The tobacco-specific nitrosamines N-nitrosonornicotine (NNN) and nicotine-derived nitrosamine ketone (NNK) may cause reproductive damage and exhibit potent carcinogenic effects in rat and human models [34,35,36,37]. Polycyclic aromatic hydrocarbons (PAHs) are immunosuppressive, carcinogenic, mutagenic, and cause reproductive damage [38,39,40,41]. Benzene is another toxic component in cigarette smoke that causes anemia, reproductive damage, bone marrow damage, and exerts carcinogenic potential [42,43,44,45,46]. Acetaldehyde is known as a skin and airway irritant and has carcinogenic properties [34,47,48]. In addition to these well-known carcinogens and mutagens, cigarette smoke also contains oxidants such as carbon monoxide, nitric oxide, hydrogen peroxide, peroxynitrate, and peroxynitrite [49]. These oxidants cause lung damage through depletion of glutathione and other antioxidants, inactivation of protease inhibitors, enhancement of the respiratory burst, and can also cause direct damage to lipids, nucleic acids, and proteins [15].

Cigarette smoking (active or passive) is the leading cause of morbidity and mortality worldwide. More than 6 million people die from cigarette-smoking-related health issues annually, including nearly 600,000 deaths due to passive or secondhand smoking. Cigarette smoking, a major risk factor for chronic lung diseases including lung cancer, COPD, and asthma [10] is a mixture of more than 4000 chemicals in its gaseous and particulate phases [11]. These chemicals include carcinogens such as benzo-α-pyrenes and acrolein, and toxicants such as acetone, nicotine, carbon monoxide, ammonia, and several oxidants (Figure 1), which may exert immunosuppressive effects [12]. Toxicants and oxidants from the gaseous phase of cigarette damage proteins, lipids, DNA, and organelles such as mitochondria and are responsible for eliciting a proinflammatory response in fetal fibroblasts [13]. For example, smoking-mediated oxidative stress resulted in DNA damage, proinflammatory cytokine release, and cell death in airway epithelial cells [14]. These oxidants cause lung damage through depletion of glutathione and other antioxidants, inactivation of protease inhibitors, and enhancement of the respiratory burst [15]. The particulate-phase toxicants also induce a proinflammatory response through activation of macrophages and modulation of epithelial cell signaling [12].

The respiratory epithelium is the primary defense site against harmful environmental agents. Smoking compromises epithelial barrier function, increases membrane permeability, and hampers mucociliary clearance [16,17,18]. Cigarette smoke exposure increases goblet cell metaplasia and mucus secretion which are one of the major pathological features in patients with COPD [19,20,21]. In addition, cigarette smoke exerts its toxic effects (e.g., apoptosis) on ciliated cells [22,23]. Cigarette smoking is associated with bacterial and viral infections in COPD patients [24], which may be associated with dysfunction of the chloride transport in epithelial cells, a feature traditionally seen in cystic fibrosis patients [25]. Animal studies suggest that cigarette smoke causes airway mucus goblet cell hyperplasia (lower doses) or metaplasia (higher doses), loss of cilia, keratinization submucosal thickening, and inflammation [26], mimicking research findings in human smokers [27,28,29].

Cigarette smoke exposure has been linked to tissue remodeling as it promotes the production of matrix metalloproteases and neutrophil elastase that contribute to connective tissue breakdown [30], a major cause of emphysema [31]. Distal airway obstruction is a common characteristic in COPD patients. It is reported that smokers with COPD have increased epithelial–mesenchymal transition (EMT, a mechanism of fibrosis) of bronchial epithelial cells compared to nonsmokers [32]. Mechanistically, fibroblasts exposed to cigarette smoke extract (CSE) released more perlecan and fibronectin, but reduced the production of proteoglycans, favoring extracellular matrix (ECM) deposition and fibrosis [33].

## 3. Lung Organoids: Introduction

Various 2D, 3D, ex vivo, and in vivo lung culture models are being used to study cigarette-smoke-induced damage. Lung organoids only need a small number of stem cells or organ-specific progenitor cells to generate self-organizing three-dimensional (3D) culture such as alveolars, airways, and lung buds [50,51]. They have been increasingly used in basic and translational research, as well as in drug discovery.

The history of lung organoids started in 1987 when Jennings et al. cultured alveolar epithelial type II (AEC2) cells in vitro. In 1991, lung tumor cells were differentiated and reorganized into an organoid structure that had similar histological attributes to the original tissue [52]. In 2009, a simple clonal sphere-forming assay was performed where mouse and human basal cells were grown without stroma. These basal cells formed “tracheospheres” with the lumen along with ciliated cells. In 2014, Jacob and coworkers developed alveolar epithelial spheroids that expressed surfactant protein C (SFTPC) and formed lamellar body-like structures [53]. In 2015, human lung organoids were cultured from hPSCs for the first time comprising all cell types of the lung including proximal airway epithelial cells, alveolar epithelial cells, and mesenchymal lineage cells [54]. This study set the foundation of lung organoid culture technology. In 2018, long-term expanding human airway organoids were generated from broncho-alveolar resections for modeling cystic fibrosis (CF). The organoids comprised basal cells, ciliated cells, goblet cells, and club cells [55].

**Figure 2:**
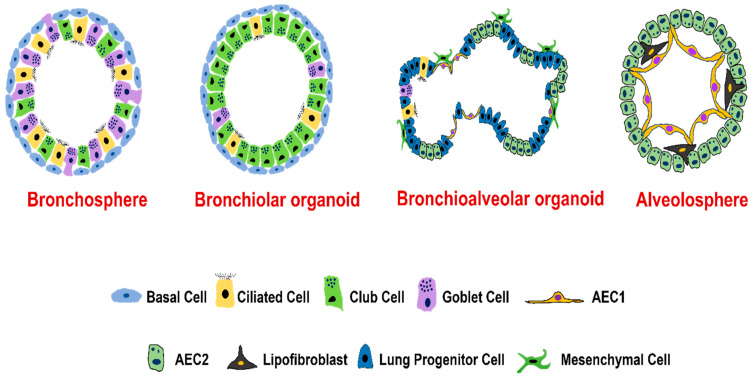
Types of lung organoids: Types of organoids derived from different parts of the lung. Airway basal cells form bronchospheres/tracheospheres made up of basal cells, ciliated cells, club cells, goblet cells. Alveolar type-2 cells along with adult human lung mesenchymal cells can generate bronchiolar organoids which consist more of club cells, ciliated cells, basal cells, and goblet cells. Bronchioalveolar organoids consist primarily of AEC2 cells, AEC1 cells, lung progenitor cells, ciliated cells, and mesenchymal cells. The alveosphere mainly contains AEC2 cells, AEC1 cells, and lipofibroblasts.

**Figure 3:**
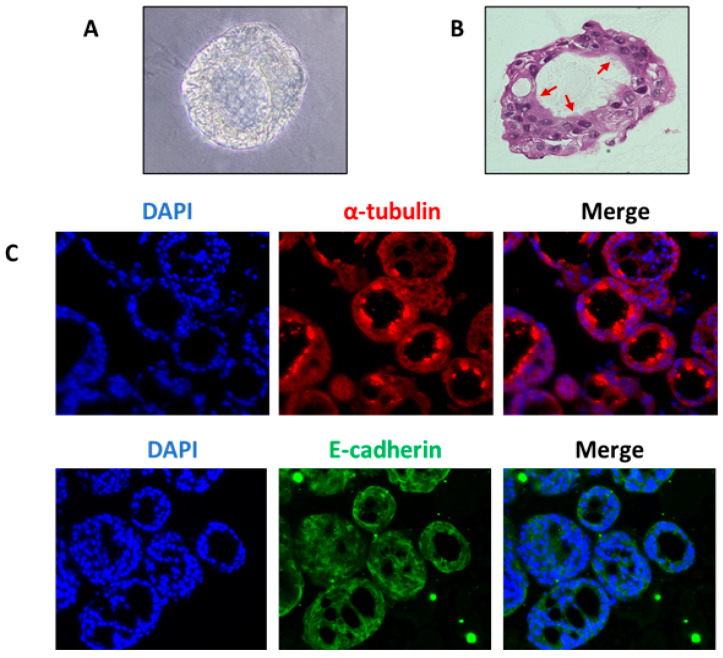
Organoid derived from human tracheobronchial epithelial cells (HTBEC): HTBECs were seeded on Matrigel and cultured in PneumaCult™ airway organoid seeding medium. Each dome consisted of 5000 cells with 90% Matrigel^®^ and 10% PneumaCult™ airway organoid seeding medium. After 7 days of expansion, cells were maintained in PneumaCult™ airway organoid differentiation medium for 21 days. After 21 days, organoids were processed for (**A**) Phase contrast microscopy (**B**) H&E staining (Red Arrows: Cilia) (**C**) Immunofluorescence staining showing tight junction of epithelial cells. (Red: α tubulin, Green: E-cadherin).

Researchers have identified several progenitor cell types from both proximal airways and distal lungs that are capable of self-organizing to form organoids (Figure 2 and Figure 3, and Table 1). Lung organoids are self-assembling structures derived from lung epithelial progenitor cells and grown on a 3D extracellular matrix, with or without feeder (mesenchymal/endothelial) cell support. The lung organoids can stimulate the developmental process of the lung and recapitulate the 3D lung structures (alveolars, airways, and lung buds) [56,57,58]. As organoids mimic in vivo physiology and genetic diversity closely, they have been considered an indispensable tool for exploring uncharted avenues in organ development, disease pathophysiology, or therapeutic biology [59]. Lung organoids can be developed from either embryonic stem cells, iPSC, or adult stem cells [60]. Organoids derived from adult stem cells are established from either a single adult stem cell or a tissue unit composed of adult stem cells by using a cocktail of growth factors and an ECM matrix, whereas organoids derived from embryonic stem cells or iPSC require a multistep process where growth factors are used to resemble the signaling pathways activated during gastrulation and organogenesis [60]. The advantages of using organoids over spheroid or 2D culture are that organoids contain multiple cell types that are found within the organ they are derived from, and they also recapitulate the cellular organization and native functionality of the organs [4]. Unlike traditional in vitro cultures, organoids are genomically stable and comprise all major cell lineages at frequencies comparable to the corresponding tissue. Another advantage of organoids is that they can be cryopreserved in biobanks and propagated/subcultured using enzymatic treatment followed by mechanical dissociation, which can then re-organize into 3D structures. In comparison to in vivo models, organoids are more amenable to all standard laboratory techniques, manipulations in signaling pathways, and genetic manipulations such as CRISPR/Cas9 [61].

Lung organoids are useful in modeling organogenesis to understand the processes associated with adult repair and homeostasis. Thus, the generation of organ-specific transplantable tissues derived from the organoids could be invaluable in regenerative treatments of the organ [56,62]. The other attractive feature of organoids is their patient specificity. Patient-specific lung organoids, due to structural and genomic similarity/identity, may predict a patient’s response toward specific drugs/treatments/therapies [51]. Organoids have helped researchers expand the study of the physiology of organs ex vivo by allowing the culture of specific cell types that were previously impractical to achieve [9].

**Table 1:** Methods to generate organoids from different cell types.

Cell Type Used	Media Composition	Matrigel Concentration	Duration of Culture	Reference
Basal cells from human trachea	Differentiation medium (Lonza) with insulin, bovine pituitary extract, hydrocortisone, GA1000, epinephrine, retinoic acid, transferrin, and triiodothyronine	25% (growth-factor-reduced) on the bottom, 5% mixed with cells	14 days	[63]
Human trachea and large airways	50:50% DMEM: BEBM (Lonza) with BEGM supplements (minus gentamycin, amphotericin, triiodothyronine, retinoic acid)	25% on the bottom, 5% mixed with cells	14 days	[64]
Alveolar epithelial cells 2 (AEC2) Supported by PDGFRα^+^ lung lipofibroblasts	MTEC^+^ media: DMEM/F12 with bovine pituitary extract, insulin, epidermal growth factor, cholera toxin, 5% fetal bovine serum, antibiotics, Y-27632 (ROCK inhibitor) for the first 2 days, and retinoic acid	1:1 ratio of Matrigel: MTEC^+^Plus media	16–17 days	[65]
Alveolar epithelial cells 2 (AEC2) supported by lung mesenchymal cells (EpCAM− Sca1^+^)	DMEM/F12 supplemented with glutamine, ITS, sodium bicarbonate, 10% newborn calf serum, antibiotics, insulin, transferrin, and selenium. FGF7, FGF10, HGF, BMP-4, TGF-β1, and PDGF-AA were added according to requirement	1:1 ratio of Matrigel: media	14–16 days	[66]
Human pluripotent stem cells	Foregut media with noggin, fibroblast growth factor 4, SB431542, CHIR99021, and other growth factors	-	15 days	[54]
Human pluripotent stem cells	Maintenance medium: DMEM/F12 (1:1) supplemented with—Primocin, FGF-2, β-mercaptoethanol, 20% knockout serum replacement Differentiation medium: IMDM/Ham’s F12 (3:1) supplemented with B27, N2, penicillin-streptomycin, bovine serum albumin, ascorbic acid, monothioglycerol, glutamax, and growth factor cocktails	100% (Branching morphogenesis)	20–25 days	[67]
Human-induced pluripotent stem cells	Directed differentiation medium: RPMI1640 media with HEPES, B27 supplement, glutamax, penicillin/streptomycin, human activin A, CHIR99021, and Y-27632	-	25 days	[68]
Human embryonic stem cells	First 3 days: RPMI1640 media with CHIR-99021 and activinA, Next 4 days: Advanced DMEM/F12 media with noggin, FGF4, SB431542, CHIR99021 After 7 days: Advanced DMEM/F12 media containing 1% fetal bovine serum	-	Up to 70 days	[69]

## 4. Types of Lung Organoids

### 4.1. Organoids from Human Airway Basal Cells

Basal cells, anchored to the basal lamina via desmosomes, are stem cells of airway epithelium that can differentiate to replenish or regenerate ciliated cells, and secretory cells including goblet cells [70]. Basal cells express genes encoding p63, KERATIN 5, INTEGRIN α6, PODOPLANIN, and the transmembrane nerve growth factor receptor (also known as p75) [2]. Organoids generated from basal cells are also known as tracheospheres (originated from trachea) or bronchospheres (originated from bronchus) [2], which are composed of basal cells and two major types of differentiated cells (goblet and ciliated cells). Like alveolar cells, the turnover of the mucociliary epithelial cells and their replacement by basal cells are very slow in normal lungs. However, during cell damage following injury by chemical agents or viral infection, there is rapid modulation in the proliferation rate of basal cells to regenerate the epithelium and restore barrier function [71]. Basal cells are grown either on Matrigel or in transwell/low attachment plates. Culture media to generate basal cell organoids from a commercial source is not clearly defined but is generally composed of supplements such as epidermal growth factor, bovine pituitary extract, insulin transferrin, selenium, cholera toxin, and retinoic acid [2]. Human basal-cell-derived organoids produce certain cytokines such as IL-6 and IL-17 that in turn influence organoid self-renewal or differentiation capabilities. IL-6 and IL-17 have been shown to increase goblet cells at the expense of losing ciliated cells, a common pathological feature of many airway diseases including asthma and COPD. Thus, basal-cell-derived organoids have been used to screen secreted proteins, small molecules, and drugs [3]. Unpublished images from our lab (Figure 3) show the presence of cilia and tight junctions in lung organoids derived from human tracheobronchial epithelial cells.

### 4.2. Organoids from Human Airway Secretory Cells

Airway secretory cells are defined as columnar, non-ciliated, non-neuroendocrine cells present in the airway epithelium. There are two major types of secretory cells: club cells and goblet cells. Club cells are non-ciliated epithelial cells found in the bronchioles. Uroplakin 3a was recently identified as a new marker for club cells. The main function of club cells is to protect the bronchiolar epithelium by synthesizing proteins such as secretoglobins (SCGB1A1, SCGB3A2) and BPIFA1 (SPLUNC1), whereas the goblet cells synthesize mucins such as MUC5AC and MUC5B to protect the lining of the lungs. Club cells also function as progenitor cells to differentiate into ciliated cells to regenerate bronchiolar epithelium [72]. Club cells display phenotypic plasticity as they can also differentiate into goblet cells in the presence of IL-13 [2]. One study demonstrated that SCGB1A1 expressing cells upon exposure to a chemotherapeutic drug, bleomycin can proliferate and differentiate into AEC1 and AEC2 cells [65]. Recently, a study identified distinct secretory cells in the human distal lung called RAS cells that differentiate into human AEC2 cells through NOTCH and WNT signaling. Interestingly, RAS cells are more abundant than SCGB1A1 and SCGB3A2 expressing club cells in the human lungs, and RAS cell to AEC2 cell differentiation is dysregulated in COPD patients [73]. However, the isolation of airway secretory cells for organoid development is mainly explored in mouse models. Two different strategies have been used to isolate the airway secretory cells for organoid culture from mouse lungs. The first approach uses fluorescence-activated cell sorting (FACS) to isolate cells based on specific cell markers. McQualter and co-workers sorted lung epithelial cells based on CD45^-^, CD31^-^, EpCAM^high^, CD49f^+^, CD104^+^, and CD24^low^ [66]. The second approach leverages lineage tracing using Scgb1a1-CreER knock-in. The other lineage tracing approach involves using *Scgb1a1-CreER* lineage cells with mouse MLg stromal cells along with a TGF-β inhibitor [64]. *Scgb1a1-Cre* labeled cells undergo a multilineage differentiation if co-cultured with Lgr5^+^ or Lgr6^+^ mesenchymal cells and exhibit bronchial, alveolar, or bronchioalveolar colonies. LGR6^+^ cells promote airway differentiation of SCGB1A1 lineage cells whereas LGR5^+^ cells promote alveolar differentiation of SCGB1A1 lineage cells [74].

### 4.3. Organoids from Human Alveolar Type II Epithelial Cells

Alveoli, the primary sites for gas–blood exchange, are the tiny air sacs situated at the end of bronchioles. Mature alveolar epithelium is composed of type I (AEC1) and type II (AEC2) epithelial cells [75]. AEC1 cells are thin flattened cells covering ~95% of the alveolar surface area. AEC2 cells, which constitute about 5% of the alveolar surface, are the cuboidal cells with distinct apical microvilli that function as stem cells for repopulating both AEC2 and AEC1 cells after lung injury [3,76]. The turnover of AEC2 cells to AEC1 happens at a very slow rate in normal lungs; however, during lung injury, there is a rapid proliferation of AEC2 cells which then differentiate into AEC1 cells to restore lung function. AEC2 cells are stem/progenitor cells that help maintain alveolar homeostasis and are thus known as the defender of alveolus [77]. With the support of mesenchymal/fibroblast/endothelial cells in the Matrigel, AEC2 cells can grow into multiple cell layer spheroids. With AEC1 cells lined along the inner lumen surface and AEC2 cells growing on the outside, these spheroids are known as alveolar organoids or alveolosphere [3,78]. It was noted that mesenchymal cells as a feeder layer promote the self-renewal of AEC2 cells [65]. When grown on top of mesenchymal cells, AEC2 cells generate an efficient alveolosphere in comparison to the alveolosphere generated using fibroblasts or endothelial cells [79].

FACS or magnetic-activated cell sorting (MACS) can be employed to isolate AEC2 cells. Cell surface markers such as HTII280 have been used to isolate human AEC2 cells. The HTII280^+^ cells develop 3D alveolar organoids with or without fibroblasts as a feeder layer [80]. Alveolar organoids have been used to study the AEC2 to AEC1 differentiation in idiopathic pulmonary fibrosis (IPF) [3], crosstalk between AEC2 and fibroblasts or macrophages. Recent application of single-cell RNA sequencing of alveolospheres helped identify a previously unknown transient state during AEC2 to AEC1 differentiation. The characteristics of the transient state correlated with that of the abnormal basal-like cells in human IPF lungs. Molecular markers observed in the identified transient state were KRT-8, CLAUDIN-4, low levels of AEC2 and AEC1 markers, TP53, TGF-β, NF-κB, and DNA damage-associated markers [3].

### 4.4. Organoids from Distal Airway Multipotent Progenitor Cells

The existence of distal airway stem/progenitor cells displaying both alveolar and airway differentiation potential has been demonstrated in several studies [73,81,82,83]. Bronchioalveolar stem cells (BASCs) were identified as the first stem cell populations in the distal lung. Cultured BASCs exhibited bonafide stem cell characteristics such as proliferative capacity and self-renewal for over eight passages [81]. BASCs express the markers for both club and AEC cells and can differentiate into club cells after naphthalene-mediated lung injury, and into AEC2 and AEC1 cells after bleomycin-induced injury [84]. Diphtheria toxin A-chain (DTA) and influenza virus infection impair the ability of BASCs to regenerate lung epithelium [83]. Studies have also shown that a single BASC can develop into a multi-lineage cell organoid when co-cultured with endothelial cells, which is dependent on endothelial thrombospondin-1. The study also highlighted the role of bone morphogenetic protein 4 (BMP4)-nuclear factor of activated T cell c1 (NFATc1)- thrombospondin-1 (TSP1) signaling axis in determining BASC differentiation to alveolar lineages [85].

### 4.5. Organoids from Human Pluripotent Stem Cells (hPSCs)

As the use of human lung tissues in research is hindered by limited access to human tissues, researchers in the past several years have focused on developing a series of protocols to differentiate hPSCs into various types of pulmonary cells. The multistep process involves differentiation of hPSCs to definitive endoderm, anterior foregut endoderm, and ventralized anterior foregut endoderm that results in a mixed population of cells including fetal-like lung and airway progenitors expressing NKX2.1 and SOX2 [86,87]. These progenitor lung lineage cells can be differentiated into the airway or alveolar cells by modulating the signaling pathways. Airway epithelial cells have been generated from iPSCs derived from chronic asthma subjects to study underlying disease pathophysiology [2]. Dye et al. in 2015 first attempted to generate hPSC-derived 3D lung organoids; however, the resulting organoids were devoid of branching morphogenesis and proximodistal specification [54]. Chen et. al., in 2017, were able to differentiate hPSCs to definitive endoderm, anterior foregut endoderm, and successfully induce them to form lung bud organoids (LBOs) [56]. Unlike earlier attempts, this model recapitulated many aspects of lung development and displayed branching morphogenesis and initial alveologenesis both in vitro and post-transplantation in vivo. Unlike alveolar and airway spheroids, which only represent the cells from a specific lung region, LBOs consist of both proximal and distal cell populations. AEC2, but not AEC1 cells, were abundantly present in LBOs, and actively uptake and secrete surfactant proteins. The LBO model is currently utilized to study pulmonary disease pathophysiology and respiratory infections [88].

### 4.6. Co-Culture Models of the Lung Organoids with Other Cell Types

Regeneration and maintenance of airway and alveolar epithelia are orchestrated by the surrounding mesenchyme and ECM in the human lung. The mesenchyme includes fibroblasts and immune cells. ECM act as a microenvironment where cells live and perform their biological functions. ECM and the mesenchyme provide complex signals to airway and alveolar epithelium and thus regulate tissue homeostasis following lung injury [89]. Lung organoids made from alveolar or epithelial progenitor cells lack stromal components such as blood vessels, connective tissue, and inflammatory cells and thus cannot recapitulate the immune responses and vascular network that mimic the interaction between tissue and vascular systems [90]. Recent reports revealed that insertion of endothelial and mesenchymal cells into lung organoids helped in multilineage communication which improved functional maturation and cellular subtype specification of lung organoids [91]. PDGFRα^+^ cells support alveolarization and epithelial development in the lung. In a study, a co-culture organoid model was developed where PDGFRα^+^ fibroblasts and AEC2 cells were grown together. The organoids formed were larger and rounder suggesting that PDGFRα^+^ fibroblasts are involved in epithelial repair [65]. PDGFRα^+^ cell lineages also act on airway epithelium. It is known that PDGFRα^+^ fibroblasts stimulate differentiation of basal cells to airway multi-ciliated cells. In another report, a 3D organoid co-culture system was generated where LGR6^+^ mesenchymal cells and pulmonary epithelial cells were grown together. Results suggested that LGR6^+^ mesenchymal cells aid in proliferation and differentiation of the airway epithelium after injury [74]. In a vascular-organoid assay, endothelial progenitor cells were found to maintain functional endothelial microvascular specificity following lung injury [92]. Macrophages are involved in inducing the inflammatory response of epithelium after lung damage. Bone-marrow-derived macrophages from normal mice or macrophages from injured mouse lungs were co-cultured with AEC2 cells. Results from that study revealed that macrophages were able to support formation of organoids derived from AEC2 cells by promoting the survival and proliferation of AEC2 cells suggesting the importance of immune cells in alveolar repair after lung injury [93].

## 5. Molecular Landscape of Epithelial Repair in Lung Organoid Models

Several epithelial repair signaling cascades are activated in response to lung injuries which have been established in traditionally used models. To ascertain the usefulness of lung organoids in disease modeling, it is important to understand the role of various signaling pathways in epithelial regeneration after lung injury (Table 2).

### 5.1. Airway Epithelial Repair

Basal cells are the key source of the primary stem cell population in the lung. After lung injury, basal cells can self-renew with pluripotent potential and differentiate into various types of cells such as basal, ciliated, or goblet. In addition to basal cells, other cell types such as club cells also play an important role as facultative stem/progenitor cells during epithelial repair. A study using the 3D co-culture organoid model showed that club cells can differentiate into alveolar lineage cells after induction with LGR5^+^ mesenchymal cells [74].

The lung organoid model can also be used to understand the molecular pathways activated in lung epithelial progenitor cells during airway epithelium repair. Notch signaling regulates the differentiation of pseudostratified columnar epithelium including basal cells. It is reported that NOTCH inhibition caused abnormal expansion of basal cells. Transgenic misexpression of NOTCH-1 in distal epithelial cells resulted in ectopic expression of club cells suggesting that notch signaling is required to maintain the club cells. Loss of regulation or abnormal expression of NOTCH is associated with the pathogenesis of various lung diseases [102]. A study with human basal cell organoids showed that differentiation of basal cells into ciliated cells was prevented by the loss of grainyhead-like transcription factor 2 (Grhl2). Upon injury, IL-1β addition to the organoid culture modulated JAG1/2 expression in ciliated cells by inhibiting the Notch pathway in secretory cells. This aided in the acquisition of differentiation plasticity. Also, transcription factor FOSL2 was identified in secretory cells which were involved in the differentiation of the secretory cells to AEC2 cells. These observations suggest the critical role of the IL-1β-NOTCH-FOSL2 axis in the fate decision of secretory cells in response to injury [94].

WNT pathway is associated with progenitor cell fate decisions during lung development [103]. WNT signaling is also involved in the repair and regeneration of airway epithelium. In a study, airway organoids were derived from the human iPSCs, and the fate of lung tissue tended to develop proximally where the WNT activity was inhibited. Another study showed that cyclic modulation of the canonical WNT pathway induced differentiation of human iPSCs through NKX2-1^+^ progenitor intermediate. This was followed by the formation of proximal airway organoids. WNT is highly activated in NKX2-1^+^ progenitor cells but can also respond to the intrinsic decrease in the WNT signaling by differentiating into proximal lineage cells instead of distal lineage cells [58]. TGF-β, a pleiotropic cytokine, is important in tissue repair. In a study, human lung fibroblasts were treated with recombinant TGF-β to induce myofibroblast differentiation. Further, fibroblasts were co-cultured with adult mouse lung epithelial cell adhesion molecule-positive cells (EpCAM^+^) to evaluate their effect on the formation of epithelial organoids in vitro. Control group lung fibroblasts promoted organoid formation in mouse EpCAM^+^ cells while the fibroblasts treated with recombinant TGF-β impaired the organoid-forming ability. RNA-sequencing analysis showed altered WNT/β-catenin signaling molecules and WNT target genes in TGF-β-treated fibroblast samples, suggesting that TGF-β-induced myofibroblast differentiation impairs the WNT/β-catenin signaling and associated components, and subsequent ability of fibroblasts to repair epithelial injury [95].

In addition, IL-6, an activator of signal transducer and activator of the transcription 3 (STAT3) pathway [104], was shown to promote the differentiation of basal cells to ciliated cells and secretory cells [96]. Autophagy may also promote airway epithelial regeneration after lung injury through reprogramming the metabolism [50]. One such study with organoid culture showed that autophagy was critical for the proliferation of airway stem-like variant club (vClub) cells during ovalbumin-induced acute inflammation. Impaired autophagy affected the glucose uptake by the vClub progenitor cells where the proliferation potential of vClub and club cells was affected due to glucose divestment and glycolysis blockade [97].

### 5.2. Alveolar Epithelial Repair

The damage-associated transient progenitors (DATPs) are a population of distinct AEC2-lineage cells involved in AEC2 to AEC1 differentiation. It was reported that DATPs were induced in the human lung organoid model after injury [105].

A study showed that a 3D alveolosphere was created using sorted AEC2 cells where AEC2 to AEC1 differentiation was dependent on the transcriptional co-activator with PDZ-binding motif (TAZ). This signaling could help in the alveolar epithelial repair after lung injury [98].

In the clonal 3D alveolar organoid model, a subset of WNT-responsive AEC2 cells was found to have transmembrane-4-L-six-family-1 as a cell surface marker which aids in the formation of large organoids containing AEC1 and AEC2 cells [106].

Another important pathway in epithelium repair is the retinoic acid (RA)/yes-associated protein (YAP) pathway. It is reported that, during the mouse development process, reduced RA signaling increased the levels of FGF10 in the distal tip mesenchyme which helped in proper airway branching [107]. RA pathway inhibition was shown to increase epithelial cell proliferation and growth in organoids derived from mice and COPD patients. Interestingly, the RA pathway inhibition also led to decreased airway and alveolar epithelial differentiation through activation of the YAP pathway and FGF signaling [99].

The transition of AEC2 cells towards AEC1 cells is an important process in alveolar epithelial regeneration and repair. A study showed the involvement of the STAT3–brain-derived neurotrophic factor (BDNF)–TrkB signaling pathway in lung repair after injury. Single-cell transcriptome analysis revealed BDNF as a STAT3 target gene. Using human and mouse alveolar organoids as a model, it was shown that STAT3–BDNF receptor tropomyosin receptor kinase B (TrkB) was a conserved pathway in epithelial repair and their dysregulation affected the alveolar epithelial regeneration after lung injury [100].

Like airway epithelial repair, the WNT signaling has a crucial role in the proliferation and differentiation of alveolar epithelial stem/progenitor cells. After lung injury, AEC2 cells are the main type of WNT-responsive alveolar epithelial cells and the maintenance of their stemness is dependent on WNT signaling. It was reported that inhibition of WNT signaling led to decreased proliferation and transdifferentiation of AEC2 to AEC1 cells [106]. It is also known that activators of WNT/β-catenin signaling (GSK3i) promote and inhibitors of WNT/β-catenin signaling inhibit the alveolar organoid formation.

Another important factor that can inhibit alveolar epithelial cell repair and regeneration is the regulation of WNT signaling through various WNT receptors. A study reported that inhibition of the FZD4 receptor reduced WNT/β-catenin activity followed by decreased cell proliferation, hampered AEC2-to-AEC1 cell transdifferentiation, and organoid formation. In addition, FZD overexpression led to the induction of WNT/β-catenin signaling with increased expression of elastogenic components [108].

Other important molecules involved in alveolar epithelial cell repair are IL-1 and TNFα, which can be induced in AEC2 cells after influenza virus infection. A study using the organoid model showed that IL-1 and TNFα increased the proliferation and differentiation capability of AEC2 cells [50]. In addition, IL-1 and TNFα were induced in AEC2 cells after influenza virus infection where AEC2 cells were involved in alveolar regeneration in the damaged area. Further, the role of the IL-1/NF-κB signaling axis was explored during AEC2 proliferation. RNA sequencing data of AEC2 cells cultured in organoids and treated with IL-1β and TNFα showed up-regulation of NF-κB1, NF-κB2, and RELB [109], suggesting a critical role of IL-1 and TNFα-mediated NF-κB in AEC2 differentiation.

IL-13, a T helper 2 (Th2) inflammatory cytokine, is associated with both obstructive and fibrotic lung diseases. IL-13 is involved in alveolar airspace enlargement, and mucus metaplasia, and induces an emphysema-like phenotype in animal models. IL-13-mediated transforming growth factor–β (TGF-β) production activates fibroblast proliferation and extracellular matrix deposition. A study from the alveolosphere organoid model combined with an in vivo model showed that IL-13 induction inhibited the differentiation of mouse and human AEC2 cells with altered morphology. Also, histological analysis suggests that the control group alveolosphere had AEC2-like cells on the outside and AEC1-like cells on the inside with multilayered architecture, but the IL-13-exposed alveolosphere did not have this organization or AEC1-like cells on the inside. Also, IL-13-treated spheres had decreased expression of the alveolar epithelial cell markers and a higher number of cells in these spheres were negative for AEC1- and AEC2-specific markers. Further, IL-13-mediated effects on AEC2 cells were dependent on STAT6 signaling from epithelial cells, but not from stromal cells [101].

The above-described airway and alveolar repair pathways in lung organoids are critical during lung repair after injury. More explanatory and mechanistic studies are needed to establish the advantages and applications of lung organoids model to understand cigarette-smoke-induced lung damage.

## 6. Application of Organoids in Cigarette-Smoking-Associated Lung Diseases

Lung organoids represent an ideal 3D in vitro model for studying the health effects of cigarette smoke as multiple types of cells are involved in response to the damage from cigarette smoke. Lung organoids display hallmark features of the human airway such as mucus secretion, ciliary beating, and regeneration which are altered during cigarette-smoke-associated pathologies. So far, few studies have been conducted using human lung organoids to determine the impact of smoking on lung biology. Organoid research is still evolving, as it was only in 2015 when researchers were successful in culturing human lung organoids from hPSCs for the first time comprising all cell types of the lung including proximal airway epithelial cells, alveolar epithelial cells, and mesenchymal lineage cells. It is premature to predict the major advantages or disadvantages of using lung organoids to study the health effect of cigarette smoke exposure. However, limited studies have demonstrated that lung organoids can mimic the complexities of the structural and functional aspects of the lung, have genomic stability, reproduce most of the characteristics of the host tissue, and contain multiple cell types, and may prove to be an ex vivo model which has the potential to overcome the limitations of traditionally used models. Here, we present current knowledge in applying lung organoids to study COPD, lung cancer, viral infection, and pulmonary fibrosis.

### 6.1. COPD

COPD is a preventable chronic lung disease manifested by chronic bronchitis and emphysema. Cigarette smoking (active or passive) is the key risk factor for COPD development. COPD is also an independent risk factor for lung cancer [110]. The development of new treatments/drugs to aid in the regeneration of the damaged or destroyed airway or alveolar tissue is mainly dependent on the preclinical models. Limited availability, high cost, and poor recapitulation of the human lung complexities remain the main concerns of using the preclinical models.

There is a dearth of reports that use lung organoids as a model to understand how cigarette smoke damages the lung. Nonetheless, limited studies using lung organoids have presented encouraging results in the aspect of mimicking the disease conditions where lung organoids successfully recapitulated some molecular cascades associated with cell damage, proliferation, differentiation, growth, repair, and remodeling comparable to cell culture and animal models. In a study employing a mouse model of chronic cigarette smoke exposure, an ex vivo alveolar organoid model was used to understand the role of a gene known as the “family with sequence similarity member 13A” (FAM13A). FAM13A mainly expressed in lung progenitor cells including club and AEC2 cells, is involved in β-catenin degradation and down-regulation of WNT/β-catenin signaling. The activation of WNT/β-catenin signaling contributes to aberrant alveolar regeneration during injury. Alveolar organoids were generated from FAM13A^+/+^ and FAM13A^−/−^ mice exposed to chronic cigarette smoke. Data from the study revealed that FAM13A deficiency upregulated WNT activation in lung epithelial cells (both club and AEC2 cells) and developed more alveolar-like organoids than FAM13A^+/+^ mice when exposed to cigarette smoke. FAM13A deficiency was also associated with increased AEC2 cell proliferation and differentiation and growth of alveolar organoids [111]. The research finding from this study is in line with the previous mouse model study where FAM13A was shown to determine the susceptibility to emphysema induced by cigarette smoke exposure, further supporting lung organoids as a valid approach to studying the pathogenesis of COPD [112].

Application of mouse lung organoid culture also revealed that prenatal cigarette smoke exposure impaired normal airway epithelial cell differentiation that was associated with induced amphiregulin (AREG) and epidermal growth factor receptor (EGFR) signaling. The organoid data showed that exposure to cigarette smoke reduced the number of ciliated cells along with decreased expression of cilia-related transcription factor forkhead box protein J1 (FOXJ1) and increased mucus-related transcription factors such as SAM-pointed-domain-containing ETS transcription factor (SPDEF) and forkhead box protein M1 (FOXM1). AEC2 cell number was found to be increased in prenatally smoke-exposed offspring. In addition, the expression of AREG was increased along with the reduced expression of epidermal growth factor (EGF) in the smoke-exposed offspring. The bronchial epithelial cell organoid model treated with AREG reduced the number of ciliated cells but increased basal cells compared to non-treated organoids. In alveolar organoids, AREG treatment resulted in a greater number of AEC2 cells [113]. This set of data supports the previous data in air–liquid interface culture of airway basal cells where cigarette-smoke-induced AREG increased EGFR activation leading to basal cells and mucous hyperplasia, impaired barrier integrity, and altered ciliated cell differentiation [114]. Together, the lung organoid model has the potential to mimic cigarette-smoke-induced molecular signaling involved in basal cell differentiation and ciliary dysfunction.

In another study [115], human or mouse CD31^-^/CD45^-^/EpCAM^+^ lung epithelial cells were co-cultured with CCL206^+^ lung fibroblasts. The purpose of this study was to identify novel receptor ligands during smoking-related lung regeneration through a transcriptomics-guided drug target discovery approach. CSE treatment to human and mouse organoids showed a reduction in size and number of organoids after 14 days. When the organoids were morphologically classified as airway and alveolar organoids, the effect of cigarette smoke exposure was more significant on the number of alveolar organoids than on the number of airway organoids. However, the size of both types of organoids was similarly reduced after 14 days of culture. Using the transcriptomics-guided drug target discovery approach, prostaglandin E2 (PGE2; target gene PTGES) and iloprost, (PGI2 analog; target gene PTGIR) were identified as the most promising targets in alveolar organoids where these genes were associated with the protection of the organoids from CSE-induced damage. In conclusion, this study supports the use of the lung organoid model to discover the novel targets associated with lung repair and regeneration in response to cigarette smoke exposure [115].

### 6.2. Lung Cancer

Lung cancer remains one of the leading causes of cancer-related mortality worldwide and cigarette smoking (active and passive) is the main risk factor for lung cancer development [116]. Carcinogens/toxicants from cigarette smoke target both distal and proximal lungs. The harmful effects of cigarette smoke carcinogens including polycyclic aromatic hydrocarbons (PAHs), aromatic amines, ethylene oxide, nitrosamines, and 1, 3-butadiene have been established in animal and human models. (Figure 1) [117]. Lung organoids derived from patients due to their capability to recapitulate cancer tissue architecture and microenvironment are now being used to understand molecular complexities and patient-specific treatment modalities [118,119].

Nicotine is one of the components of cigarette smoke which promotes proliferation, migration, and invasion of cells in vitro and promotes tumor growth and metastasis in vivo. The effect of nicotine on the stemness of cancer cells was analyzed where A549 and H1650 non-small-cell lung cancer cell lines were treated with 2 μM nicotine and sphere formation capacity was analyzed. It was found that nicotine treatment induced the sphere formation capacity and migration potential of both the cell lines after 10 days of treatment suggesting that nicotine increases the self-renewal potential of lung cancer cells [120]. This data correlated with the previous mice study where nicotine treatment increased stemness and epithelial–mesenchymal transition of human umbilical cord mesenchymal stem cells which was crucial for tumor growth and promotion [121].

In a mouse lung organoid culture study, it was reported that long-term (3 months) intermittent cigarette smoke exposure induced severe emphysema [122,123] along with greater adenocarcinoma-related changes in comparison to long-term continuous cigarette smoke exposure. In this study, female mice expressing GFP in AEC2 cells were exposed to mainstream cigarette smoke through nasal inhalation. Furthermore, AEC2 cells derived from the mice were cultured with fibroblasts in stem cell 3D organoid/colony-forming assays. Results suggested that both intermittent- and continuous-cigarette-smoke-exposed cells formed more colonies in comparison to non-treated cells. However, AEC2 cells derived from the intermittent-cigarette-smoke-exposed mice produced more colonies than those derived from mice with continuous cigarette smoke exposure. Intermittent cigarette smoke exposure induced alveolar stem cell expression of genes (e.g., CPT1A) related to the fatty acid oxidation (FAO) pathways. The colony formation potential of AEC2 cells was significantly reduced in intermittent-CS-exposed mice when treated with etomoxir, an inhibitor of the FAO regulator CPT1A [124]. This study revealed that the lung organoid model can mimic the molecular complexities and responses of intermittent and continuous cigarette smoke exposure and may provide new insights into mechanisms of cigarette-smoke-induced lung cancer [124].

### 6.3. Respiratory Viral Infections

Smoking is one of the major risk factors for respiratory infections. Respiratory viral infections have been implicated in disease exacerbations [125,126]. Cigarette smoke disturbs the normal function of the immune system where smoke inhibits airway inflammatory and antiviral responses to influenzas viruses [127,128,129,130]. Viruses mainly target airway epithelial cells and AEC2 cells in the respiratory epithelium [67]. Lung organoid models have been increasingly utilized to study host–virus interactions due to their ability to recapitulate the respiratory epithelium. In a study, lung organoids derived from hPSCs were infected with the human parainfluenza virus. Whole-genome sequencing data suggested that the parainfluenza virus was able to replicate in the organoids. Measles virus infection to these organoids resulted in syncytium formation [67]. In another study, human lung PSC-derived organoids infected with the respiratory syncytial virus (RSV) demonstrated epithelial alterations, cytoskeleton rearrangement, and syncytia formation and thus recapitulating the in vivo pathologies [56]. The lung organoid model is also being used to study the severe acute respiratory syndrome coronavirus 2 (SARS-CoV-2). Lung bud tip organoid-derived bronchioalveolar-like cells infected with SARS-CoV-2 showed surfactant protein C-positive AEC2-like cells with increased type I/III interferon expression and viral titers. Similarly, lung organoids were used to understand the infectivity of influenza viruses in humans. In this study, human proximal differentiated airway organoids containing ciliated, goblet, club, and basal cells were infected with a highly infective H1N1pdm virus. The data indicated that the lung organoids model successfully mimicked the host–viral interactions and recapitulated the morphological/functional/molecular changes associated with lung damage [131].

Application of lung organoids to study the effect of smoking on respiratory viral infection, especially SARS-CoV-2 infection, is urgently needed. Two-dimensional models can only partially recapitulate the complexity of the alveolar space and epithelium and cannot be cultured for long periods [132]. Animal models are still the prime models used in respiratory virus research. However, in today’s world, emerging viruses such as the SARS-CoV-2 virus can develop epidemic- or pandemic-like scenarios very rapidly. As animal model research is time-consuming, it can delay the research to understand disease pathology/drug development. Lung organoids can mimic lung complexities and can be developed rapidly [131]. Thus, using the lung organoid model may provide new mechanisms for cigarette-smoke- or vaping-associated respiratory infections. We anticipate that more lung organoid studies will be performed and published by the scientific community in the next year or two to demonstrate if smoking alters lung immunity against viral infections.

### 6.4. Idiopathic Pulmonary Fibrosis (IPF)

IPF is a chronic, progressive, and fibrotic disease characterized by scarring of the lung parenchyma. Cigarette smoke is a significant risk factor for IPF and can impact the survival of patients with IPF [133]. Although the exact mechanism by which cigarette smoke contributes to IPF is not known, recent evidence suggests that oxidative stress may play a role [133]. IPF is mainly described by the uncontrolled activation of fibroblasts. In response to tissue injury/damage, fibroblasts release various profibrotic, proangiogenic, and proinflammatory components including transforming growth factor-β (TGF-β), and promote disease progression [134]. The bleomycin-induced mouse model and other models have some similarities with human IPF settings, but they can’t completely mimic the pathophysiology of IPF. Lung organoids can recapitulate the IPF associated changes, physiologically mimic the microenvironment, organization of fibrotic tissue, and the response against cigarette-smoke-induced lung damage. Thus, the cigarette smoking model of lung organoids may further aid in the understanding of IPF.

The organoid model is now being used to recapitulate some key features of IPF. Human PSC-derived mesenchymal cell organoids treated with TGF-β demonstrated elevated expression of α-SMA and collagen I along with organoid contraction that was related to fibrotic progression [135]. Strikoudis et al. used the CRISPR/Cas9 gene-editing technique in lung organoids derived from hPSCs to study the role of Hermansky–Pudlak syndrome (HPS)-associated interstitial pneumonia (HPSIP) in IPF. They found that IL-11 released from HPS1^−/−^ ESC-derived organoids may promote fibrogenesis [136].

A damaged alveolar/distal epithelium is observed in patients with IPF. To understand the molecular mechanisms of distal lung diseases, human iPSC-derived alveolar epithelial organoids treated with a fibrosis cocktail (FC) including TNFα, PDGF-AB, TGFβ, and LPA reduced the expression of surfactant protein-C and elevated the expression of vimentin and senescence-associated genes responsible for epithelial injury and reprogramming. Further, the expression of KRT17 and KRT8 was increased, which are crucial markers for dysregulated epithelial repair [137]. In conclusion, this study demonstrated that iPSC-derived alveolar epithelial cells were able to recapitulate several key features of IPF such as epithelial injury, reprogramming, induction of cellular senescence-associated genes, and extracellular matrix production, thus making the lung organoid model suitable for drug discovery and disease modeling [137].

Overall, the use of lung organoids in studies mentioned in this section provided a better understanding of cigarette-smoke-induced molecular and phenotypic changes which could not have been possible with simpler models such as 2D cell culture or spheroids. Other models such as ALI can recapitulate the respiratory physiology but cannot mimic the spatial structure of the trachea. Thus, lung organoids that contain various differentiated cell types with complex tissue architecture and more closely resemble in vivo tissues and functionality, may provide a realistic model to understand cigarette smoke associated lung pathologies. Unfortunately, very few studies have reported the detailed molecular mechanisms altered by cigarette smoke in the organoid models and, thus, more studies are needed to establish this model as a better alternative to understand various lung pathologies.

## 7. Limitations of the Organoid Model

Organoids are utilized to study organ and tissue development and pre-clinical drug screening and understand the pathophysiology of various lung diseases. Despite the promising aspects of the organoid model, like other models, they also have some limitations.

- Organoids are mostly derived from iPSCs. The cells from iPSCs are immature, imitate the second trimester of fetal development, and have similar gene expression profiles to the embryo, thus making it difficult to mimic adult diseases [138].
- Organoids are heterogeneous and have several variabilities that appear at many stages such as (1) between genotypes and different starting cell lines, (2) between batches of the organoids from the same starting material, (3) between different organoids in the same culture, and (4) between areas of a single organoid.
- The organoid model may be devoid of some of the cell types. During organogenesis, cells from different organs/origins (such as bone marrow and neural crest cells) are also present that aid in organ development which is not present in the organoid model. [139].
- Due to the lack of vascularization, the in vitro growth potential of organoids is restricted. As undefined mouse-derived ECM is required for organoid culture, batch-to-batch variation is a major concern. Also, solidified gel-ECM affects the penetration and availability of nutrients, drugs, and stimuli under investigation, causing variations in the results. Despite being heterogeneous in comparison to 2D cultures, the variable size, cell ratios, and morphology of organoids make them difficult for phenotypic screening. This limitation also makes it difficult to incorporate microsensors for critical control and functional parameters such as fluid pressure, oxygen, glucose, flow, cell migration, and barrier integrity. To overcome this limitation, lung-on-a-chip devices are now being developed where cells are cultured in a uniform manner and have a defined orientation [140].
- In addition to vasculature, the other important factor that affects organoid development and differentiation is the culture media. To date, none of the media used in organoid culture are chemically defined. The organoid media also contain some complex components such as bovine pituitary extract (BPE) or fetal bovine serum (FBS). Oxygen tension and glucose levels are also not tested in this system. Thus, we still do not have a defined list of growth factors, metabolites, or small molecules required for the renewal and differentiation of organoids [2]. Some recent studies reported chemically defined media with growth factors for the establishment of the organoid culture. Although, the ECM components and the mechanical forces need further assessment in detail [2,55,80,141].
- The organoid model is a single-organ model and cannot completely mimic the physiological niche provided by neural, immune, and stromal cells. Thus, how long these organoids can survive is a major limitation that makes them less complicated as compared to in vivo models [142]. To overcome these limitations, the lung organoid model is co-cultured with endothelial cells and immune components to make organoids better mimic the in vivo human lung physiology. Another approach being used is to combine organoids with an organ-on-a-chip which can prove to be an ideal research tool for high-throughput screening of drugs, regenerative biology, and understanding smoking-related disease pathophysiology [143,144]. Furthermore, using stem cell-derived organoids with an organ-on-a-chip approach can help develop patient-specific disease models that can open new avenues in personalized therapy.

Further research is needed to overcome these limitations to make the organoid model a better and indispensable tool that can provide an alternative to the costly and time-consuming in vivo models.

## 8. Other Models to Study the Effect of Smoking on Lung Health

**Figure 4:**
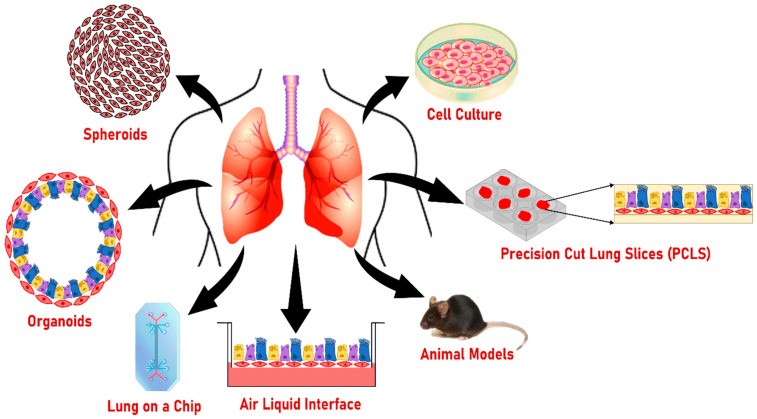
Models to study the effect of smoking on lung diseases: Types of different 2D in vitro (cell culture)*,* 3D in vitro (Spheroids, ALI, and lung-on-a-chip), ex vivo (PCLS, organoids), and in vivo (animal) models to study the effect of smoking on lung disease pathophysiology.

Various 2D and 3D models are being used in smoking studies to understand lung disease pathophysiology (Figure 4 and Table 3). The advantages and disadvantages of these models are briefly described below:

### 8.1. Two-Dimensional Cultures

Immortalized cell lines/cancer cells/continuous cell lines have been used in toxicology studies including smoking studies for a long time. Cancer cells such as the A549 cell line are easy to culture and have an extended life span. A549 cells are lung adenocarcinoma cells isolated from the lung tissue of a white, 58-year-old male [59]. Agraval et al. showed that cigarette smoke exposure to A549 cells elevated the activity and expression of MMP-2 and MMP-9 which in turn regulated EMT-associated changes [145]. BEAS-2B and 16HBE14o- are the other immortal cell lines commonly used in inhalation studies [146]. BEAS-2B epithelial cells are isolated from normal human bronchial epithelium derived from autopsies of noncancerous individuals and 16HBE14o- is a human bronchial epithelial cell line derived from a 1-year-old male patient and immortalized with the origin-of-replication-defective SV40 plasmid (pSVori-). A study showed that BEAS-2B treated with CSE triggered cell death, induced autophagy, and phosphorylation of JNK [147]. Cigarette smoke exposure to 16HBE14o- disrupts the cell–cell contacts followed by an impairment of barrier function [148]. Traditional 2D cell culture models are economic, easy to handle, and less time-consuming. This makes them a suitable option for high-throughput screening of drugs. However, the disadvantage of using 2D cell culture models for smoking studies is the limited differentiation potential of primary or immortalized cell lines and the absence of a microenvironment that is present in the human lungs. Lung organoids recapitulate the 3D organizational structure (such as alveolars, airways, and lung buds) [50], exhibit better self-renewal and differentiation, and are comprised of different types of cells present in the microenvironment [2]. Thus, lung organoids could be used to study smoking-associated lung pathologies.

### 8.2. Air–Liquid Interphase Culture (ALI)

In submerged 2D in vitro cell culture models, the physiological attributes of airway mucosa are absent [149]. In addition, exposure or inhalation toxicology studies with submerged culture do not mimic or precisely represent the cell–cell and cell–stimulant interactions [150,151,152,153]. ALI culture is a 3D in vitro cell culture model which allows the airway cells to differentiate into a pseudostratified cell layer, consisting of goblet/secretory cells, ciliated cells, and basal cells [154], mimicking the in vivo airway epithelial arrangement. One such study shows that human bronchial epithelial cells (HBEC) grown and differentiated at ALI for 7, 14, 21, or 28 days, when exposed to CSE, affected the barrier function, increased the goblet and club cells, and decreased the ciliated cells [155]. Due to its physiological relevance to in vivo models, ALI models are now being widely used in smoking studies that have shown the detrimental effects of tobacco smoking on airway epithelial barrier function, basal cells, goblet cells, club cells, and ciliated cells [155]. However, the ALI model also has some limitations. The ALI model is static, has limited microenvironment control, and cannot completely recapitulate the naïve airway epithelium. ALI requires a considerable number of cells to initiate the culture which is difficult to obtain in patient-specific studies [156]. Lung organoids can be derived from a smaller number of cells, reproduce most of the characteristics of the host tissue, and recapitulate the lung structure and function [51].

### 8.3. Lung on a Chip

Organ on a chip or lung on a chip is a microengineered microfluidics cell culture device created with microchip-manufacturing methods [157]. Organ on a chip can either be a single cell or multiple cell type in nature and has a microfluidics device for the continuous supply of nutrients and removal of waste. These devices can mimic the cellular architecture, microenvironment, and tissue–tissue interfaces of an organ and thus can be used to study various lung diseases such as lung cancer, COPD, and asthma [158,159]. Techniques such as lithography-based microfabrication, thermoplastic technique, and 3D bioprinting are used for the fabrication of the lung on a chip model.

Application of cigarette smoke-exposed human ‘small airway-on-a-chip’ containing differentiated, mucociliary bronchiolar epithelium and an underlying microvascular endothelium suggested that smoke exposure increased oxidative stress as indicated by upregulated heme oxygenase-1 (HMOX1) gene expression and ciliary dysfunction. Increased expression of serine protease genes TMPRSS11E and TMPRSS11F was observed which can be correlated with airspace enlargement, extracellular matrix degradation, and emphysema in COPD patients [160]. Another study using a lung-on-a-chip model with pulmonary epithelial cells and vascular endothelial cells showed that CSE enhanced the release of IL-6 and TNF-α and dysregulated air–blood barrier function [161]. These data also correlate with previous data from the mice model, where mice were subchronically exposed to mainstream tobacco smoke (MTS) and expression of HMOX-1 was found to be up-regulated. In this study, levels of IL-6 antigens were also induced 4-fold compared to matched controls after 12 weeks of exposure to MTS [162]. This explains that the lung-on-a-chip model can recapitulate the complexities of mice lungs and mimic the organ function and response.

The lung-on-a-chip model involves advanced technological tools where special instruments are required to control fluid and air flow. Challenges in fabrication, operational control, and complex design and manufacturing processes limit the use of this model on a large scale. Organoids can recapitulate the lung structure and function, but they lack the vasculature and immune component and do not have microenvironment control either. Combining both models could provide us with a better and more reliable model to understand lung disease pathologies where cigarette smoke is the main risk factor. In one such study, alveolar feeder-free organoid cultures were derived from emphysema patients followed by expansion of the cells on a chip-S1 organ chip. The cells were able to retain AEC2 cell markers and this combination culture method was able to recapitulate and model the (diseased) alveolar compartment [143].

### 8.4. Precision-Cut Lung Slices (PCLS)

PCLS is an ex vivo model which can be used to study the lung health effect of environmental risk factors and the therapeutic efficacy of various compounds or drugs. It comprises three-dimensional lung tissue slices that retain most types of the lung cells (biologically active), complex microarchitecture, anatomical architecture, functional response to stimuli observed in native lung tissue, and other organ-specific features, such as tissue homeostasis, metabolic activity, and immunological functions up to a certain extent [163,164]. During PCLS preparation, a lung lobe obtained from a donor with or without lung disease is inflated with low-melting agarose to retain the structure of airways, alveoli, and other compartments for slicing using a vibratome [165]. Due to its ability to cut thousands of slices from a single lung lobe, the PCLS model has become more popular in studies aimed at understanding the pathophysiology of lung diseases such as viral infection, COPD, asthma, and lung cancer [166]. Human PCLS exposed to CSE was shown to increase proinflammatory cytokines MMP-9, IL-1α, and IL-1β and cause tissue damage which correlated with the previous mice data, where cigarette smoke treatment for 1 month increased the expression of MMP-9 and the specific inhibitor of MMP-9 ameliorated the small airway remodeling and emphysema-associated changes [167]. The main limitations of PCLS models are related to the lack of recruitment of nonresident immune cells and the short duration of viability reported to be only up to 28 days [165]. Also, the PCLS model cannot recapitulate the lung development process and, thus, cannot be used to study the effect of smoking on neonatal stage lung development. Lung organoids, on the other hand, can be cultured for a longer time [55] and recapitulate some of the developing processes of neonatal lungs [113].

### 8.5. Spheroids

Spheroids serve as another 3D cell culture model formed by cell self-aggregation to mimic the structural characteristics of tissues and microtumors [168]. Spheroids are either self-floating or seeded on 3D bioscaffolds without the need to use an extracellular matrix for cell attachment [169]. As they are composed of patient-derived cells or tumor-derived cells, spheroids are useful to study cell interactions in lung progenitor cell differentiation and therapeutic response in cancer and pulmonary fibrosis [170]. Few reports using the 3D airway spheroid model to study the smoking effect are available. A study reports that BEAS-2B cell-derived spheroids treated with air pollutants and diesel exhaust particles resulted in an EMT-like phenotype characterized by increased collagen 1A1 and β-catenin expression but decreased adhesion molecules such as E-cadherin and ZO-1 [74]. However, the spheroid model has some limitations including lack of vasculature, uncontrolled cell ratios, and aggregate size, which makes it unable to mimic the organ function such as host–pathogen interactions [72].

### 8.6. Animal Models

Animal models are one of the top choices for translational research and drug development of human lung diseases [171]. Both mouse and rat models have been utilized to extensively study the acute and chronic effects of tobacco smoking, and recently determine the health effect of vaping [172,173]. Overall, these studies revealed mild to severe damaging effects of smoking and vaping on cell integrity, inflammation, and tissue structure such as emphysema. However, the data obtained from the animal models cannot always reflect the complexity of human genetics and disease pathobiology [171,174]. For example, human subjects did not show any beneficial effects by using the drugs that were declared safe in animal studies. Isoprenaline, a drug approved for the treatment of asthma based on animal studies, caused over 3500 deaths in Britain alone, highlighting the safety concerns of these drugs tested on animal models [175].

## 9. Conclusions and Perspectives

Smoke from cigarettes or other sources (e.g., vaping) is known to cause detrimental effects on the lung mainly characterized by reversible or irreversible changes such as inflammation, fibrosis, and emphysema. The lung epithelial lining is the first site of defense against various environmental insults including cigarette smoke and electronic cigarettes [176]. Exposure to cigarette smoke is associated with mucus hypersecretion, squamous metaplasia, and impaired mucociliary clearance. These epithelial changes cause small-airway remodeling where dysregulated EMT and abnormal proliferation and differentiation of epithelial cells lead to subepithelial fibrosis [32,177,178].

Various in vitro and in vivo models are being used to study the effect of smoking on the pathophysiology of lung diseases. ALI culture is used widely for smoking studies as an alternative for 2D cell line models. Although, it is reported that the maximum pollutant dose absorbed by the ALI model remains low due to the technical limitations when compared to the ambient pollutant concentration [149]. With PCLS models, the main limitation is the availability of the tissue and the viability concerns in response to cigarette smoke treatment. Although animal models have been used for years in inhalation and infection studies, they also have some limitations such as interspecies variability, cost, and relevance to clinical translation. Thus, there is an urgent need for developing new models and strategies that can overcome some of these limitations.

Recently, 3D stem-cell-derived organoids have emerged as an alternative ex vivo model that recapitulates some key features of lung diseases in a dish. Organoids recapitulate the structural and functional aspects of the lung including cell–cell and cell–niche interactions during homeostasis, development, and diseases [179]. Thus, lung organoids serve as a powerful tool to model lung development, regeneration, and diseases. The main advantage of using lung organoids as a model is that they are physiologically relevant. They show the characteristic attributes of the airways including regeneration, mucus secretion, and cilia beating. They exhibit the self-renewal and differentiation capacities of the stem cells and form an organized structure after differentiation. Although the absence of non-epithelial cells including the immune cells and the endothelial cells fails to create the vasculature and recapitulate the architecture of the lung, studies have shown that the introduction of mesenchymal cells into organoid culture induced renewal and differentiation of the stem cells [65,180,181]. Also, macrophage addition to 3D organoid cultures supported the growth of the AEC2 cells [93]. These reports suggest that the introduction of immune cells, mesenchymal cells, and endothelial cells will aid in recapitulating the complexities of the lung architecture and will improve our understanding of the stem cells, their microenvironment, and the interplay between these factors [3].

To understand the functional heterogeneity of lung organoids, isolation and purification of subpopulations of stem and progenitor cells and stromal support cells with different surface markers are required. This will also help in developing new protocols to study the maturation and differentiation of organoids. Personalized/patient-derived organoids such as cystic fibrosis (CF) organoids or lung-tumor-derived organoids have proven to mimic the disease. Thus, using personalized organoids would help in understanding the individual’s response to therapies/treatments. Organoids are also used as a source of transplantable tissue for cell replacement therapies [182,183]. Organoids developed from cells freshly isolated from the late stage of diseased lungs will increase our understanding of the biomarkers and molecular pathways activated at the later stages of lung disease including smoking-related lung pathologies. The use of gene-editing techniques such as CRISPR/Cas9 will open new avenues to study genetic diseases where the role of individual genes in self-renewal and differentiation of human lung stem cells can be explored. In the future, optimized organoid technology will be useful to model various attributes of lung development, regeneration, and diseases. This will enhance our ability to identify novel biomarkers and treatment modalities in various lung pathologies where smoking is a common risk factor.

## Figures and Tables

**Table 2:** Molecular pathways and functions in organoid models during epithelial repair.

Model	Species	Cells Used to form Organoids	Signaling Pathway	Biological Function	Link between Biological Function and Disease	References
Lung organoid model to study airway epithelial repair						
Airway organoids	Human	Secretory cells and AEC2 cells	IL-1β-NOTCH-FOSL2 axis	Acquisition of differentiation plasticity; differentiation of the secretory cells to AEC2 cells	To understand the role of NOTCH and IL-1β in repair and regeneration signaling pathways in response to lung injury	[94]
Airway organoids	Human	iPSCs	WNT signaling	Repair and regeneration of airway epithelium	To understand the role of WNT signaling in human airway patterning which can aid in lung disease modeling	[58]
Adult lung epithelial progenitor cell organoid	Human and mouse	Human lung fibroblasts with adult mouse lung epithelial cell adhesion molecule-positive cells (EpCAM^+^)	TGF-β- WNT/β-catenin signaling	TGF-β activation impairs fibroblast ability to support adult lung epithelial progenitor cell organoid formation	To understand the aberrant mesenchymal–epithelial signaling during COPD and IPF pathophysiology	[95]
Airway organoids	Mouse	Basal cells	IL-6-STAT3 pathway	Differentiation of basal cells to ciliated cells and secretory cells	To evaluate the IL-6/STAT3 signaling in multi-ciliogenesis and airway repair process in response to lung injury	[96]
Airway organoids	Mouse	Airway stem-such as vClub cells	Glucose uptake through endocytosis/recycling of GLUT1	Autophagy-regulated lung epithelial proliferation and regeneration	To understand the role of autophagy in repair of injured epithelium in response to allergens or other types of lung injury	[97]
Lung organoid model to study alveolar epithelial repair						
Alveolar organoids	Mouse	AEC2 cells	PDZ-binding motif (TAZ)	Lung alveolar epithelial cell differentiation	To understand the role of lung alveolar epithelial cell differentiation in response to lung injury during the pathogenesis of IPF	[98]
Adult lung organoids	Human and mouse	Primary mouse and human lung epithelial cells (airway and alveolar epithelial cells)	Retinoic acid (RA)–yes-associated protein (YAP) pathway	Balances adult distal lung epithelial progenitor cell growth and differentiation	To understand the role of the retinoic acid–yes-associated protein pathway in endogenous lung regeneration during COPD pathogenesis	[99]
Alveolar organoids	Human	AEC2 cells	STAT3–BDNF–TrkB signaling pathway	Alveolar–epithelial regeneration and repair	To evaluate the role of the STAT3–BDNF–TrkB signaling pathway in alveolar–epithelial regeneration during viral infections and acute respiratory distress syndrome	[100]
Alveolar organoids	Human	AEC2 cells	WNT/β-catenin signaling	Proliferation and transdifferentiation of AEC2 to AEC1 cells and maintenance of stemness	To understand the regulation of WNT signaling in the alveolar epithelial progenitor cells of COPD patients and discover new treatment strategies	[50]
Alveolar organoids	Mouse	AEC2 cells	IL-1 -TNFα—NF-κB signaling pathway	Increased alveolar proliferation, differentiation, regeneration, and repair	To understand the role of IL-1/TNFα-NF-κB signaling axis in tissue recovery following injury (e.g., influenza-induced injury)	[50]
Alveolosphere organoids	Human and mouse	AEC2 cells	IL-13/STAT6 pathway	Decrease expression of the alveolar epithelial cell markers; sustain the inflammatory response	To understand the IL-13-mediated chemokine and inflammation-driven responses in COPD and pulmonary fibrosis pathogenesis	[101]

**Table 3:** Models used in smoking studies.

Model	Advantages	Disadvantages
Cell lines	Less expensive. Useful in high-throughput screening of drugs. Easy to use. Less time-consuming.	Genetic alterations. Limited barrier activity. Finite life span. Inadequate differentiation potential. Absence of stromal–vascular–inflammatory cell components.
ALI	High reproducibility. Easy to use. Patient-specific. Compatible with high-throughput screening.	Lack of in vivo architecture. Cannot completely mimic the naïve airway epithelium. Limited microenvironment control.
Lung on a chip	Controlled microenvironment. Mimics organ function. Presence of vasculature. Alveolar–capillary interface.	Certain cell types are missing. Experts required. Non-standardized protocols. Complex design and manufacturing process. Difficulties and expenses associated with fabrication and operational control. Time-consuming. Requires advanced technology. Not useful in high-throughput screening.
PCLS	Intact cellular architecture. Active biological processes. Represents multiple cell types.	Availability of subjects/tissue. Cell viability.
Spheroids	Recapitulate cell–cell interactions. Easily reproducible. Physiologically relevant. Strong cell–ECM interactions.	Difficult to control the aggregate size and cell ratios. Not able to mimic organ function. Lack of vasculature. Complex culture protocol.
Animal models	Physiological similarities with humans. Presence of stromal–vascular–inflammatory cell components.	Costly. Time-consuming. Safety and ethical concerns. Anatomical differences between humans and mice. Interspecies variability.
Organoids	Recapitulate structural and functional aspects of the lung. Contain multiple cell types. Genomically stable. Preservable in biobanks. Short-term study. Reproduce most of the characteristics of the host tissue. Compatible with high-throughput screening. Suitable for gene editing.	Lack of vascularization and immune cells. Variable size, cell ratios, and morphology of organoids. Limited microenvironment control. Limited control of the spatiotemporal distribution of nutrients and metabolites.

## Data Availability

All data relevant to the study are included in the article.

## Abbreviations

COPD	Chronic obstructive pulmonary disease
ALI	Air liquid interface
PCLS	Precision cut lung slices
hPSCs	Human pluripotent stem cells
ASCs	Adult stem cells
iPSCs	Induced pluripotent stem cells
EMT	Epithelial–mesenchymal transition
CSE	Cigarette smoke extract
NNN	N-nitrosonornicotine
NNK	Nicotine-derived nitrosamine ketone
ECM	Extracellular matrix
PAHs	Polyaromatic hydrocarbons
AEC2	Alveolar epithelial type II
AEC1	Alveolar epithelial type I
SFTPC	Surfactant protein C
CF	Cystic fibrosis
HTBEC	Human tracheobronchial epithelial cells
FACS	Fluorescence-activated cell sorting
MACS	Magnetic-activated cell sorting
IPF	Idiopathic pulmonary fibrosis
BASCs	Bronchioalveolar stem cells
BMP4	Bone morphogenetic protein 4
NFATc1	Nuclear factor of activated T cell c1
TSP1	Thrombospondin-1
LBO	Lung bud organoid
Grhl2	Grainyhead-like transcription factor 2
STAT3	Signal transducer and activator of the transcription 3
DATPs	Damage-associated transient progenitors
TAZ	Transcriptional co-activator with PDZ-binding motif
YAP	Yes-associated protein
FGF	Fibroblast growth factor
BDNF	Brain-derived neurotrophic factor
TrkB	Receptor tropomyosin receptor kinase B
Th2	T helper 2
TGF-β	Transforming growth factor–β
FAM13A	Family with sequence similarity member 13A
AREG	Amphiregulin
EGFR	Epidermal growth factor receptor
FOXJ1	Forkhead box protein J1
FOXM1	Forkhead box protein M1
SPDEF	SAM pointed domain containing ETS transcription factor
PGE2	Prostaglandin E2
FAO	Fatty acid oxidation
RSV	Respiratory syncytial virus
SARS-CoV-2	Severe acute respiratory syndrome coronavirus 2
HPSIP	Hermansky–Pudlak syndrome (HPS)-associated interstitial pneumonia
BPE	Bovine pituitary extract
HBEC	Human bronchial epithelial cells
HMOX-1	Heme oxygenase-1
MTS	Mainstream tobacco smoke
RA	Retinoic acid
FC	Fibrosis cocktail
DTA	Diphtheria toxin A-chain
EGF	Epidermal growth factor
CPT1A	Carnitine palmitoyltransferase 1A
FBS	Fetal bovine serum
